# Effects of Night-Time Use of Rotigotine on Nocturnal Symptoms in Parkinson's Disease

**DOI:** 10.1155/2015/475630

**Published:** 2015-10-21

**Authors:** Francesc Vallderiola, Yaroslau Compta, Javier Aparicio, Jaume Tarradellas, Gabriel Salazar, Josep María Oliver, Antonio Callén, Tania Delgado, Fritz Nobbe

**Affiliations:** ^1^Parkinson's Disease and Movement Disorders Unit, Neurology Service, Institut de Neurociències Hospital Clínic, University of Barcelona, Barcelona, Catalonia, Spain; ^2^Institut d'Investigacions Biomèdiques August Pi I Sunyer (IDIBAPS), Barcelona, Catalonia, Spain; ^3^Epilepsy Unit, Department of Neurology, Hospital Clínic, Barcelona, Catalonia, Spain; ^4^Neurology Service, Clínica Dexeus, Barcelona, Catalonia, Spain; ^5^Neurology Service, Hospital de Terrassa, Barcelona, Catalonia, Spain; ^6^Cap Clìnic de Neurologìa, Hospital Universitario Reus and Department of Medicine and Surgery, Universitat de Tarragona, Tarragona, Catalonia, Spain; ^7^Neurology Service, Parc Sanitari Sant Joan de Déu, Sant Boi, Barcelona, Catalonia, Spain; ^8^Neurology Service, Hospital Parc Taulí de Sabadell, Catalonia, Spain; ^9^Clínica Juaneda, Palma de Mallorca, Balearic Islands, Spain

## Abstract

*Objectives.* This open-label study assessed the efficacy and safety of exclusive night-time administration of transdermal rotigotine in patients with nocturnal and early morning PD symptoms. *Methods.* Patients with PD and nocturnal and early morning symptoms received transdermal rotigotine patches (2–16 mg/24 h) applied in the evening and removed in the morning for 3 months. Sleep disturbance was assessed with modified Parkinson's Disease Sleep Scale (PDSS-2). Other outcomes included a pain visual analogue scale (VAS) and short-form Parkinson's Disease Questionnaire (PDQ-8) for quality of life. *Results.* 74 patients completed treatment in this study. At the end of treatment, PDSS-2 total score had improved by mean 10.9 points from baseline (*p* < 0.001). All three PDSS-2 domain scores (sleep disturbances, nocturnal motor symptoms, and nocturnal symptoms) were also significantly improved by 41%, 56%, and 48%, respectively (*p* < 0.001). VAS-pain score decreased from 3.2 to 2.3 (*p* < 0.001). PDQ-8 score decreased from 23.8 to 18.1 (*p* < 0.001). The most frequently reported adverse events included nausea (9%), anxiety (4%), and dizziness (4%). *Conclusions.* Night-time administration of transdermal rotigotine is an effective and well tolerated treatment for nocturnal symptoms in patients with PD.

## 1. Introduction

Sleep disorders are common in patients with Parkinson's disease (PD), occur throughout the course of PD, and are a major contributor to reduced quality of life (QOL) [1, 2]. Insomnia, which is one of the most common sleep disturbances in PD, can be a consequence of other nocturnal PD symptoms, such as akinesia, dystonia, pain, tremor and nocturia, and comorbid sleep-related disorders, such as restless legs syndrome (RLS) [2, 3]. There is evidence for a dopaminergic component to sleep disorders in PD [4], but the effects of dopaminergic stimulation on sleep and wakefulness in PD are complex and not well understood. Dopaminergic therapies may improve nocturnal sleep in patients with PD, by reducing nocturnal motor and nonmotor symptoms and by influencing intrinsic sleep mechanisms [3, 5, 6].

It is generally agreed that the most effective treatment for PD symptoms is levodopa, but levodopa has a short plasma half-life of 60–90 min, and the duration of benefit eventually declines [7]. Pulsatile dopaminergic stimulation and levodopa-associated motor fluctuations may contribute to nocturnal symptoms of PD. Therefore, continuous dopamine receptor stimulation during the night with long-acting dopamine agonists may alleviate these symptoms [8, 9].

Rotigotine is a nonergoline dopamine receptor agonist administered via a transdermal patch that facilitates constant release and stable plasma concentrations of the drug over 24 hours, thereby avoiding pulsatile stimulation of dopamine receptors [9, 10]. In the European Union, the rotigotine transdermal patch is approved as monotherapy in early PD and in combination with levodopa throughout the course of PD [11]. It is also approved for the symptomatic treatment of moderate-to-severe idiopathic RLS in adults [11].

Studies investigating the effect of transdermal rotigotine on nocturnal symptoms have shown that 24-hour administration has beneficial effects on sleep and other motor and nonmotor symptoms [5, 6, 12]. We aimed to determine if the effects of night-time rotigotine on the nocturnal symptoms of PD were similar to that observed with 24-hour transdermal rotigotine and whether this exclusive night-time administration of rotigotine minimized the risk of treatment-related adverse events (AEs). To do this, the current open-label study was designed to assess the clinical efficacy and safety of night-time administration of transdermal rotigotine for treating nocturnal symptoms in patients with PD.

## 2. Methods

### 2.1. Study Design

This prospective, open-label, single-arm study was conducted at seven Spanish centres between November 2012 and November 2013. Eligible patients had PD diagnosed according to UK PD Society Brain Bank criteria [13], with unsatisfactory control of nocturnal and early morning symptoms, as assessed by the treating physician during routine clinical practice. No changes in PD treatment were permitted in the 28 days before entering the study. Patients were excluded if they had moderate or severe cognitive impairment that would interfere with daily living activities (assessed by clinical interview and routine cognitive examination), had Hoehn and Yahr Stage V disease, had a history of dopaminergic treatment-related AEs, or did not have bothersome nocturnal and early morning symptoms.

The study was conducted according to the principles of the Declaration of Helsinki and complied with good clinical practice as described in the ICH Guidelines for Good Clinical Practice. The study protocol and consent form were approved by the Independent Ethics Committee of the Hospital Clínic, University of Barcelona. Written informed consent was obtained from all participants.

### 2.2. Treatment

Rotigotine was prescribed and titrated in accordance with standard clinical practice. Patients were instructed to apply the rotigotine patch each evening, keep it on overnight, and remove it in the morning. Over 1–8 weeks, treatment was titrated to optimal dose at which the treating physician and patient felt that nocturnal symptoms of PD were adequately controlled, starting with a patch that released 2 mg/24 h or 4 mg/24 h, increasing in weekly increments of 2 mg/24 h up to a maximum of 16 mg/24 h.

### 2.3. Assessments

Patients were assessed before initiation of rotigotine (baseline) and at the end of treatment. The primary objective was to assess the changes from baseline in nocturnal symptoms, patient QOL, pain perception, and Clinical Global Impression of Severity (CGI-S) scores at 3 months. The secondary objective was to assess the tolerability of rotigotine.

The effect of rotigotine on sleep and nocturnal disability was measured using a modified version of 15-item Parkinson's Disease Sleep Scale (PDSS-2) [14], with a total score ranging from 0 to 60, where higher scores indicate greater impairment. Three PDSS-2 domain scores (“disturbed sleep,” “motor symptoms at night,” and “PD symptoms at night”) were calculated by summing individual item scores in groups of five for a maximum score of 20. Changes in patient QOL were assessed by asking patients to complete short-form Parkinson's Disease Questionnaire (PDQ-8) [15] (scores range from 0 to 100, with higher scores reflecting worse QOL). Changes in pain level were assessed using a visual analogue scale (VAS) that ranged from 0 (no pain) to 10 (severe pain). The final efficacy assessment was the CGI-S, whereby the investigator rated the patient on a 4-point scale (not at all ill, slightly ill, moderately ill, and very ill).

The frequency and severity of AEs were recorded and the relationship between each reported AE and rotigotine was assessed.

### 2.4. Statistical Analyses

No formal sample size calculation was undertaken for this study, but it was planned to recruit 10–12 patients per treating neurologist, up to a maximum of 100–120 patients. It was expected that 80% of patients would complete treatment.

Efficacy analyses were performed for all patients who received at least one dose of rotigotine and had at least one efficacy assessment. Safety analyses were performed for all patients who received at least one dose of rotigotine.

Descriptive statistical analyses were performed with SPSS 19.0 software (IBM Corporation, Armonk, NY, USA, 2010). Changes in PDSS-2 scores were analyzed using the paired Student *t*-test, while changes in PDQ-8, VAS, and CGI-S scores were analyzed using the Wilcoxon test (this nonparametric test was used due to the lower range of scores for the domain scores). Quantitative data are presented as means and standard deviations. Qualitative data are presented as absolute numbers and percentages. Associations between changes in efficacy scales and patient gender and reasons for initiating nocturnal rotigotine were evaluated using the Mann-Whitney *U* and Kruskal-Wallis tests, respectively. Associations between changes in efficacy scales and patient age, time from diagnosis, Hoehn and Yahr disease stage, and baseline scores were evaluated using Spearman's or Pearson's correlation coefficients. All analyses were two-tailed with significance level set at ≤0.05.

## 3. Results

### 3.1. Patient Disposition and Baseline Characteristics

Of 81 patients included in the study, 74 completed treatment. Rotigotine was withdrawn due to AEs in 5 patients. One patient made their own decision to withdraw treatment. Another patient was arbitrarily withdrawn by another physician.

Patients were a mean 71.5 years old (54% male) and were all receiving concomitant therapy with other dopaminergic agents (Table 1). The most common reason for initiating rotigotine was difficulty maintaining sleep (88% of patients), followed by nocturia (69%). Over half the patients initiating rotigotine had difficulty falling asleep, nocturnal/early morning akinesia, restless legs, or vivid dreams. Overall, 69% of patients were receiving concomitant medication for comorbid conditions, the most common of which were psychiatric, gastrointestinal, and cardiovascular disorders (data not shown).

### 3.2. Rotigotine Dose

The rotigotine starting dose was 2 mg/24 h (*n* = 65) or 4 mg/24 h (*n* = 16). Final dose was 2 mg/24 h (*n* = 3), 4 mg/24 h (*n* = 36), 6 mg/24 h (*n* = 9), and 8 mg/24 h (*n* = 19).

### 3.3. Efficacy

#### 3.3.1. Parkinson's Disease Sleep Scale

**Figure 1:**
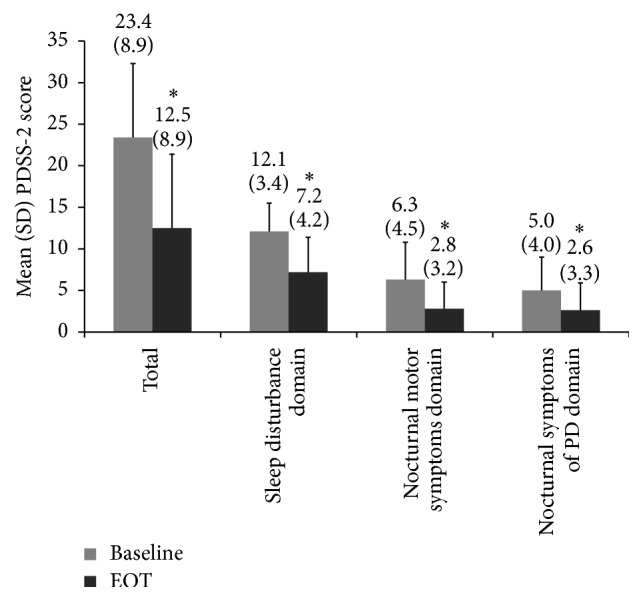
Change in PDSS-2 total score and domain scores from baseline after 3 months of nightly treatment with rotigotine transdermal patch in the efficacy population (*n* = 74). EOT, end of treatment; SD, standard deviation. ^*∗*^ *p* < 0.001 versus baseline.

PDSS-2 total score significantly improved by mean 10.9 points (47%) from baseline after 3 months (*p* < 0.001, Figure 1). All three PDSS-2 domain scores were also significantly improved at the end of treatment (*p* < 0.001); the sleep disturbances domain score improved by 4.9 points (41%), the nocturnal motor symptoms domain score improved by 3.5 points (56%), and the nocturnal symptoms of PD domain score improved by 2.4 points (48%).

The higher the baseline PDSS-2 total score, the greater the reduction in the score after 3 months (*r* = 0.57, *p* < 0.0001). This was also the case for the sleep disturbance domain score (*r* = 0.47, *p* < 0.001), the nocturnal motor symptoms domain score (*r* = 0.75, *p* < 0.001), and the nocturnal symptoms of PD domain score (*r* = 0.65, *p* < 0.001). Patients who had difficulty falling asleep had significantly greater improvements in PDSS-2 total score (*p* = 0.003) and the sleep disturbance domain score (*p* = 0.006), compared with patients who had other reasons for starting rotigotine. Patients who initially had leg and arm pain had significantly better improvements in PDSS-2 total score (*P* = 0.02) and the nocturnal symptoms of PD domain score (*p* < 0.001), and patients with restless legs had a significantly better improvement in the nocturnal motor symptoms domain score (*p* < 0.001). Improvements in PDSS-2 scores did not significantly differ according to whether or not patients had difficulty maintaining sleep, nocturia, nocturnal/early morning akinesia, or vivid dreams as the primary reason for starting rotigotine.

#### 3.3.2. Other Efficacy Outcomes

PDQ-8 and VAS-pain scores significantly improved after 3 months of treatment (*p* ≤ 0.001, Table 2); PDQ-8 score decreased by 5.6 points (23%); and VAS-pain decreased by 0.9 points (28%). Although the quantitative change in CGI-S score was not statistically significant, the number of patients judged to be severely or moderately ill on CGI-S decreased from 47 (64%) to 38 (51%) at 3 months, with a proportionate increase in patients who were assessed as not at all or mildly ill (*p* = 0.001).

Larger improvements were significantly associated with higher baseline scores for all three scales: PDQ-8 (*r* = 0.50, *p* < 0.001), VAS-pain (*r* = 0.47, *p* < 0.001), and CGI-S (*r* = 0.32, *p* = 0.005). Patients who initially had pain in legs and arms had significantly greater improvements in PDQ-8 (*p* < 0.005) and VAS-pain (*p* < 0.001) than patients with other reasons for starting rotigotine.

### 3.4. Safety and Tolerability

Overall, 24 (30%) patients reported 42 AEs; the most common AE was nausea (Table 3). Of the 42 AEs, 28 were considered probably related to rotigotine and 14 were considered to be possibly related. Most AEs were of mild to moderate intensity. There were 16 severe AEs in 10 (12%) patients. Severe AEs comprised pain, dizziness, nausea, and vomiting in 2 patients each and reduction in visual acuity, hallucination, confusion, rash, flatulence, hypotension, vertigo, and blurred vision in 1 patient each. Rotigotine was withdrawn because of the severity of AEs in 11 patients (during the 3-month trial period for 5 patients and after the final visit for further 6 patients), while dosage was reduced in 7 patients. The only AEs resulting in rotigotine withdrawal or dose reduction in ≥2 patients were nausea (*n* = 4), anxiety (*n* = 3), pain (*n* = 2), and dizziness (*n* = 2).

## 4. Discussion

Night-time administration of transdermal rotigotine resulted in significant improvements in sleep and nocturnal disability in patients in PD. These changes were evidenced by significant reductions in PDSS-2 total score and disturbed sleep, motor symptoms at night, and PD symptoms at night domain scores. Other efficacy assessments revealed significant improvements in pain, QOL, and CGI-S.

The effects of nocturnal administration of rotigotine on sleep and nocturnal disability in this study were assessed using the PDSS-2, which is a modified version of the original PDSS that was developed to better reflect treatment effects on nocturnal disabilities [6]. The significant improvement in sleep on the PDSS-2 is consistent with the result of a previous open-label trial that used the original PDSS and in which rotigotine was administered for the full 24-hour period [5]. Furthermore, a randomized, double-blind study of 24-hour rotigotine therapy (RECOVER) has reported a 4.3-point improvement in PDSS-2 total score versus placebo (*p* < 0.0001), where significant improvements in all three PDSS-2 domain scores were also reported relative to placebo [6]. Our results are, thus, consistent with existing evidence.

The 47% improvement in PDSS-2 total score after 3 months of treatment with rotigotine in the current study compared favorably with the improvement in PDSS-2 relative to baseline after 3 months of 24-hour administration of rotigotine in RECOVER and the improvement in PDSS score in the previous open-label trial [5, 6]. This suggests that the exclusive night-time application of transdermal rotigotine did not limit efficacy with regard to PDSS total scores.

To our knowledge, this is the third but largest open-label study to investigate night-time application of transdermal rotigotine for nocturnal symptoms in patients with PD. Both previous studies had much smaller sample-size (5 and 10 patients) and showed PDSS improvements relative to baseline of 18% and 45%, respectively [9, 16]. The differences in efficacy between these two previous studies may have been a consequence of shorter duration of treatment (1 versus 4 months) and a cohort with less advanced PD in the study with the smallest PDSS improvement [16]. The 45% reduction observed in the study by Canesi and colleagues compared favorably with the 31.5% reduction in RECOVER, which may stem from inclusion of patients with long disease duration (mean 17.4 years versus 4.6 years in RECOVER) [9]. This theory is not supported by our results, whereby a 47% improvement was observed in PDSS-2 score in patients with mean duration since diagnosis of 5.6 years. However, our patients were relatively old (mean age: 71.5 years versus 65 years in RECOVER and 60 years in Canesi and colleagues study), and, as in RECOVER, most were receiving levodopa. Additionally, in our study patients with the highest baseline PDSS-2 total scores benefited most from rotigotine. Overall, these observations suggest that older patients with longer disease duration and severe nocturnal symptoms may particularly benefit from rotigotine.

A* post hoc* analysis of the RECOVER data has shown that only 13% of the improvements in PDSS-2 scores could be related to improvements in motor symptoms [3]. Compared with patients who had other reasons for starting therapy with rotigotine in this study, patients who had difficulty falling sleeping had significantly greater improvements in PDSS-2 total score and the sleep disturbance domain score, patients who initially had leg and arm pain had significantly better improvements in PDSS-2 total score and the nocturnal symptoms of PD domain score, and patients with restless legs had a significantly better improvement in the nocturnal motor symptoms domain score. As illustrated by a significant improvement in the VAS, pain was significantly improved after 3 months of night-time treatment with rotigotine. In a* post hoc* analysis of the 15 individual PDSS-2 items in RECOVER, 10 were significantly improved versus placebo, including “difficulty falling asleep,” “urge to move arms or legs,” “uncomfortableness and immobility,” “restlessness of arms or legs”, “pain in arms or legs”, and “muscle cramps in arms or legs” [6]. Taken altogether, these observations suggest that patients with insomnia and problems with pain and/or restless limbs may find night-time use of transdermal rotigotine particularly beneficial.

PDQ-8 score decreased by 5.6 points (23%) in this study and by 6.9 points (22%) in RECOVER [6], reflecting similarly improved QOL in both studies. In this study, Spearman's analysis revealed significant associations between the improvement in PDQ-8 and the improvements in PDSS-2 total and the three domain scores (data not shown), reflecting the importance of sleep disorders in predicting poor QOL in patients with PD.

Rotigotine was generally well tolerated. The most frequent AEs included nausea and dizziness and the frequencies of these AEs were lower than in RECOVER [6]. Application site reactions were also one of the most frequent AEs associated with 24-hour application of the rotigotine transdermal patch in RECOVER, occurring in 15% of patients treated with rotigotine [6]. Application site reactions (20%) and nausea (15%) were similarly common in the open label trial in which rotigotine was administered to 54 patients over the full 24-hour period. In contrast, there was only one application site reaction reported in the current study. In general, it seems possible that night-time application of the rotigotine patch may reduce potential for such AEs relative to the standard 24-hour application.

Dopaminergic therapies may be associated with the onset or aggravation of excessive daytime sleepiness (EDS) in PD patients, but it is recognized that EDS is common in PD and is also likely to be related to the disease as well [17, 18]. Also of concern are the impulse-control disorders (ICD) associated with dopamine agonists [19, 20]. Although neither EDS nor ICD was specifically investigated in RECOVER, there were no reports of EDS (but two rotigotine-treated patients had sleep attacks) or ICD in this study [6]. However, in RECOVER, 4% of rotigotine-treated patients had a positive result on the Minnesota Impulsive Disorder Interview and an additional patient had positive findings of compulsive sexual behavior on the structured psychiatric interview [6].

The relevance of the results of this study is limited by the open-label design, relatively small sample size, and short duration. However, the agreement of our findings with results of the randomized, placebo-controlled RECOVER study and the persistence of such effect for up to 1 year in a subsequent open-label extension [6, 21] lend external consistency to the current study results. As is the case in previous trials [5, 6], there are no sleep laboratory data to objectively measure sleep outcomes. Ideally, a randomized, placebo-controlled trial of exclusive night-time use of the rotigotine transdermal patch that includes sleep laboratory assessment is required to confirm the results observed.

The results of this study suggest that night-time administration of transdermal rotigotine is effective for control of nocturnal symptoms in patients with PD. Rotigotine induced a reduction in nocturnal disability and minimized the emergence of undesirable dopaminergic effects and application site reactions. Administering rotigotine at night-time was well tolerated, with fewer AEs observed than during traditional 24-hour/day administration. Older patients with relatively advanced PD and severe nocturnal symptoms may particularly benefit from night-time use of transdermal rotigotine, as may patients with insomnia and problems with painful or restless limbs.

## Figures and Tables

**Table 1:** Baseline demographic and clinical characteristics.

Characteristic	Safety population (*n* = 81)
Age, years	71.5 ± 8.6
Gender	
Male, *n* (%)	44 (54.3)
Female, *n* (%)	37 (45.7)
Time since first diagnosis, years	5.6 ± 5.2
Hoehn and Yahr stage, *n* (%)	
I	17 (21)
II	33 (40.7)
III	27 (33.3)
IV	4 (4.9)
PDSS-2 total score	23.3 ± 8.8
PDQ-8 score	24.2 ± 19.2
VAS-pain score	3.2 ± 2.6
CGI-S score	3.7 ± 0.6
Concomitant dopaminergic medication, *n* (%)	81 (100)
Levodopa preparations	70 (86.4)
Dopamine agonists	32 (39.5)
MAO-B inhibitors	31 (38.3)
Entacapone	2 (2.5)
Major complaints of patients who received nocturnal rotigotine, *n* (%)	
Difficulty maintaining sleep	71 (87.7)
Nocturia	56 (69.1)
Difficulty falling asleep	46 (56.8)
Nocturnal/early morning akinesia	42 (51.9)
Restless legs	42 (51.9)
Vivid dreams	41 (50.6)
Pain in arms and legs	31 (38.3)

Unless otherwise specified, data are mean ± standard deviation.

CGI-S, Clinical Global Impression of Severity; MAO-B, monoamine oxidase B; PDQ-8, short-form Parkinson's Disease Questionnaire; PDSS, Parkinson's Disease Sleep Scale; VAS, visual analogue scale.

**Table 2:** PDQ-8, VAS-pain, and CGI-S scores at baseline and after 3 months of months of nightly treatment with rotigotine transdermal patch in the efficacy population (*n* = 74).

Assessment scale	Mean ± SD score	
	Baseline	EOT
PDQ-8	23.8 ± 18.6	18.2 ± 17.7^a^
VAS-pain	3.2 ± 2.5	2.3 ± 2.4^a^
CGI-S	3.7 ± 0.6	3.5 ± 0.7

^a^ *p* < 0.001 versus baseline.

CGI-S, Clinical Global Impression of Severity; EOT, end of treatment; PDQ-8, short-form Parkinson's Disease Questionnaire; SD, standard deviation; VAS, visual analogue scale.

**Table 3:** Most frequently reported^a^ AEs during 3 months of nightly treatment with transdermal rotigotine in the safety population (*n* = 81).

Adverse event	Number (%) of patients		
	Overall	Relation to treatment	
		Probable	Possible
Nausea	7 (8.6)	6 (7.4)	1 (1.2)
Anxiety	3 (3.7)	3 (3.7)	0
Restlessness	3 (3.7)	2 (2.5)	1 (1.2)
Dizziness	3 (3.7)	1 (1.2)	2 (2.5)
Pain	2 (2.5)	2 (2.5)	0
Rash	2 (2.5)	2 (2.5)	0
Vomiting	2 (2.5)	1 (1.2)	1 (1.2)

^a^Reported by at least 2 patients overall; patients could report ≥1 AE.
